# Automated Axial Right Ventricle to Left Ventricle Diameter Ratio Computation in Computed Tomography Pulmonary Angiography

**DOI:** 10.1371/journal.pone.0127797

**Published:** 2015-05-22

**Authors:** Germán González, Daniel Jiménez-Carretero, Sara Rodríguez-López, Kanako K. Kumamaru, Elizabeth George, Raúl San José Estépar, Frank J. Rybicki, Maria J. Ledesma-Carbayo

**Affiliations:** 1 Madrid-MIT M+Visión Consortium, Research Laboratory of Electronics, Massachusetts Institute of Technology, Cambridge, Massachusetts, United States of America; 2 Biomedical Image Technologies, Universidad Politécnica de Madrid & CIBER-BBN, Madrid, Spain; 3 Applied Imaging Science Laboratory, Brigham and Women´s Hospital, Boston, Massachusetts, United States of America; 4 Surgical Planning Laboratory, Brigham and Women´s Hospital, Boston, Massachusetts, United States of America; Rutgers University -New Jersey Medical School, UNITED STATES

## Abstract

**Background and Purpose:**

Right Ventricular to Left Ventricular (RV/LV) diameter ratio has been shown to be a prognostic biomarker for patients suffering from acute Pulmonary Embolism (PE). While Computed Tomography Pulmonary Angiography (CTPA) images used to confirm a clinical suspicion of PE do include information of the heart, a numerical RV/LV diameter ratio is not universally reported, likely because of lack in training, inter-reader variability in the measurements, and additional effort by the radiologist. This study designs and validates a completely automated Computer Aided Detection (CAD) system to compute the axial RV/LV diameter ratio from CTPA images so that the RV/LV diameter ratio can be a more objective metric that is consistently reported in patients for whom CTPA diagnoses PE.

**Materials and Methods:**

The CAD system was designed specifically for RV/LV measurements. The system was tested in 198 consecutive CTPA patients with acute PE. Its accuracy was evaluated using reference standard RV/LV radiologist measurements and its prognostic value was established for 30-day PE-specific mortality and a composite outcome of 30-day PE-specific mortality or the need for intensive therapies. The study was Institutional Review Board (IRB) approved and HIPAA compliant.

**Results:**

The CAD system analyzed correctly 92.4% (183/198) of CTPA studies. The mean difference between automated and manually computed axial RV/LV ratios was 0.03±0.22. The correlation between the RV/LV diameter ratio obtained by the CAD system and that obtained by the radiologist was high (r=0.81). Compared to the radiologist, the CAD system equally achieved high accuracy for the composite outcome, with areas under the receiver operating characteristic curves of 0.75 vs. 0.78. Similar results were found for 30-days PE-specific mortality, with areas under the curve of 0.72 vs. 0.75.

**Conclusions:**

An automated CAD system for determining the CT derived RV/LV diameter ratio in patients with acute PE has high accuracy when compared to manual measurements and similar prognostic significance for two clinical outcomes.

## Introduction

Pulmonary embolism (PE) is a common disease with an incidence greater than 1 per 1000 in the U.S. and an overall three month mortality rate of approximately 15% [1]. Several treatments are available for PE, ranging from anticoagulant therapy to surgical embolectomy. Optimal patient management relies on proper risk stratification that uses clinical judgment supplemented with objective data based on laboratory values and estimation of right ventricular size and function [2]. Morbidity and mortality from PE results from overload on the right ventricle. Uncompensated pressure can result in right ventricular dilation and hypokinesis, which can lead to right ventricular failure and ultimately death.

Clinical suspicion of PE is most often confirmed with Computed Tomography Pulmonary Angiography (CTPA) [3,4] that also images the cardiac chambers and enables measurement of the Right Ventricular to Left Ventricular diameter ratio (RV/LV). Introduced more than 15 years ago [5], this metric predicts mortality in patients with severe PE [6–10].

Quantification of the RV/LV ratio by CT can use chamber volumes or diameter measurements made on either four chamber reformatted views or axial images alone [11–14]. These methods are subject to a significant inter-reader variability [15] and are moderately time consuming for the radiologists, since several caliper positions need to be evaluated, measured and the ratio finally computed and reported. One method to simplify the assessment of the right ventricle is to report enlargement subjectively [16]. However, this method is expected to depend even more on the experience of the radiologist.

This study describes and tests a fully automated computer aided detection (CAD) system that analyzes the ventricles from the CTPA images used for PE diagnosis, and outputs the axial RV/LV diameter ratio. While axial ventricular diameters do not represent true ventricular diameters, their prognostic value for patients suffering from acute PE has been shown not to be inferior to the prognostic value of ventricular ratios obtained in four chamber reformatted images [12].

The CAD system produces automatically an objective and reproducible axial RV/LV diameter ratio. In this study we focus in the design and retrospective validation of the output of the CAD system and leave the evaluation of its integration in the clinical workflow and the evaluation of the effort required to correct its output as future work.

## Materials and Methods

### Evaluation Database

The Brigham and Women’s Hospital (BWH) Institutional Review Board (IRB) approved this HIPAA-compliant retrospective cohort study; written informed consent was waived since patient data was analyzed anonymously. All consecutive CTPA examinations performed at BWH (large, urban, teaching hospital) from February 2009 to November 2009 were examined and 200 studies positive for acute PE were identified. The mean age was 60 ± 16 years (range 22–89), 113 patients were female and 87 were male. Fifty-eight patients had central PE; 13 patients had saddle emboli and 45 patients had emboli in either the main left, main right or both main pulmonary arteries. Systolic blood pressure was available for 176 patients. Massive PE, as defined in [17], was identified in 3 patients.

The axial RV/LV diameter ratio of this cohort of patients used as reference standard in the current study has been previously reported [13]. That report compared different manual methods to estimate the RV/LV diameter ratio, whereas the current project analyzes the performance of a CAD system when computing automatically axial RV/LV diameter ratios. DICOM data from two of the 200 identified CTPA studies were not accessible and therefore not available for this analysis. Thus, the final analyses included 198 patients. All cases were considered of adequate quality to perform PE detection.

The Social Security Death Index was used to confirm patient death. The cause of death was determined by consensus of three observers, after independent review of autopsy reports, death certificates, and electronic medical records. Thirty-two deaths within 30 days were reported and, of those, 22 were PE-related deaths. Additionally the administration of intensive therapies during the initial hospitalization was obtained from hospital electronic medical records: 13 patients who did not die within 30 days received intensive therapies and 5 of them needed combined treatments. The therapeutic interventions were: thrombolysis (n = 7), vasopressor therapy for systemic arterial hypotension (n = 4), mechanical ventilation (n = 3), catheter intervention or surgical embolectomy (n = 4). No patient was lost to follow-up. Two outcomes are defined to test the predictive value of the automated axial RV/LV diameter ratio: PE-related death within 30 days (n = 22) and a composite outcome consisting on the PE-related death within 30 days or the need for intensive therapies (n = 35).

### Image Acquisition

CTPA images were acquired in craniocaudal direction using 16-slice or 64-slice scanners (Siemens Healthcare, Erlangen, Germany) and reconstructed at 1.0 mm slice thickness. Scanning parameters included 80–120 kVp and approximately 200 effective mAs. All patients received 75–100 mL of contrast media that contained 370 mg iodine/mL either in the form of iopromide (Bayer HealthCare, Berlin, Germany) or iopamidol (Bracco Diagnostics, Princeton, NJ, USA). Contrast was administered using a power injector at a rate of 3 mL/s. Image acquisition was timed using bolus tracking on the main pulmonary artery with a threshold attenuation of 80 HU.

### Manual assessment of RV/LV diameter ratio

A radiologist with 6 years of experience in cardiovascular imaging reviewed the images. For each case, the RV/LV diameter ratio was calculated on axial images using a Vitrea fX 3.1 workstation (Vital Images, a Toshiba Medical Systems Group, Minnetonka, MN, USA). RV and LV diameters were defined as the largest distance between the surface of the interventricular septum and the endocardium within all axial slices in the heart region. These RV and LV maximum diameters may be found at different craniocaudal levels. Another radiologist with 2-years of experience repeated the same measurements independently in 30 randomly selected scans to assess inter-observer variability. In nine patients, the CT images were separately reviewed to estimate the time that it takes a radiologist to compute the axial right ventricular to left ventricular diameter ratio.

### CAD Method

**Fig 1:**
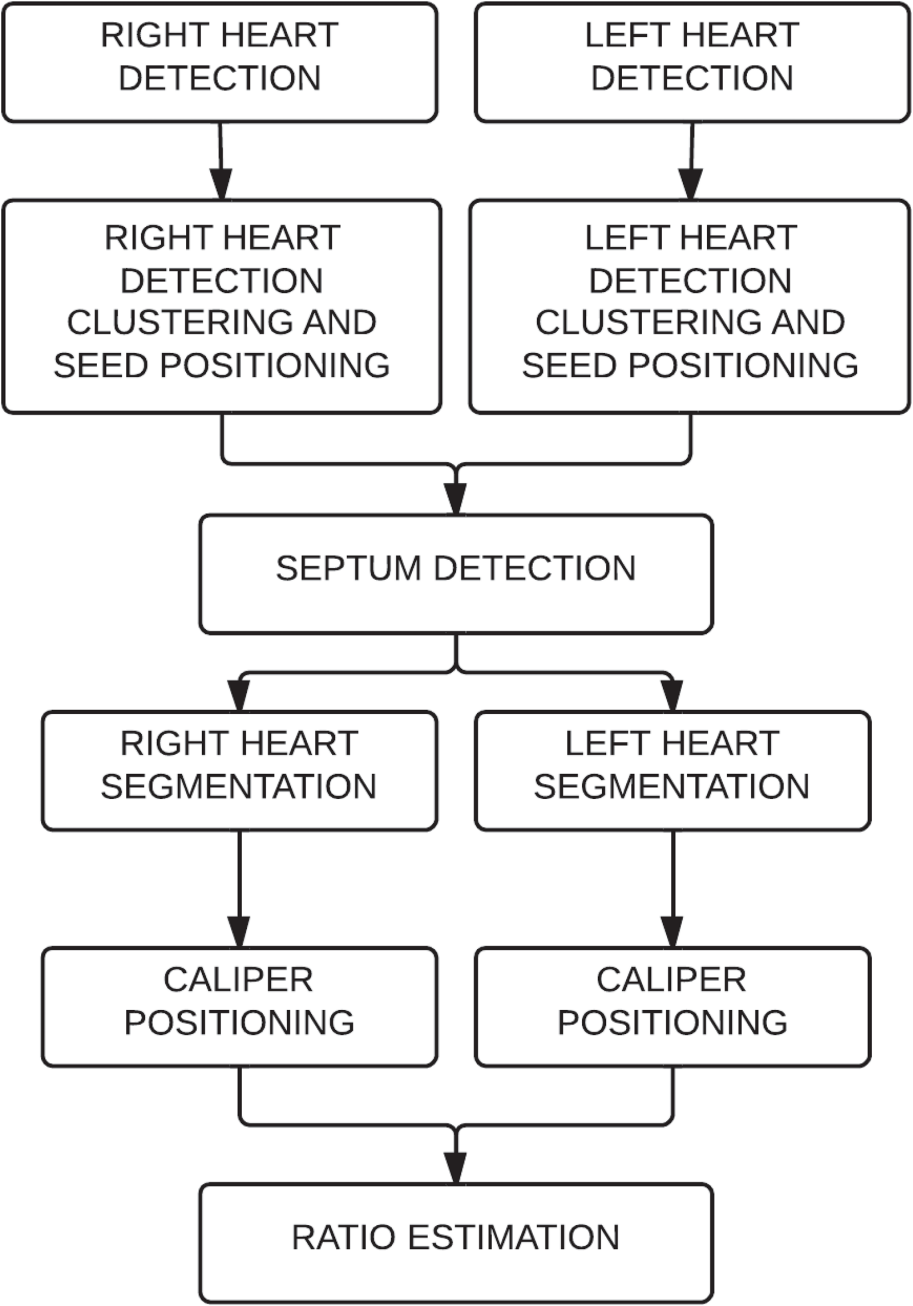
Algorithm description. First, the right ventricle and the left ventricle are detected on each axial slice. The detections are clustered to position seeds for further segmentation. The seeds are used to detect the septum. Using the seeds and the septum, the ventricles are segmented and the calipers positioned. Finally the right ventricular to left ventricular axial diameter ratio is estimated.

**Fig 2:**
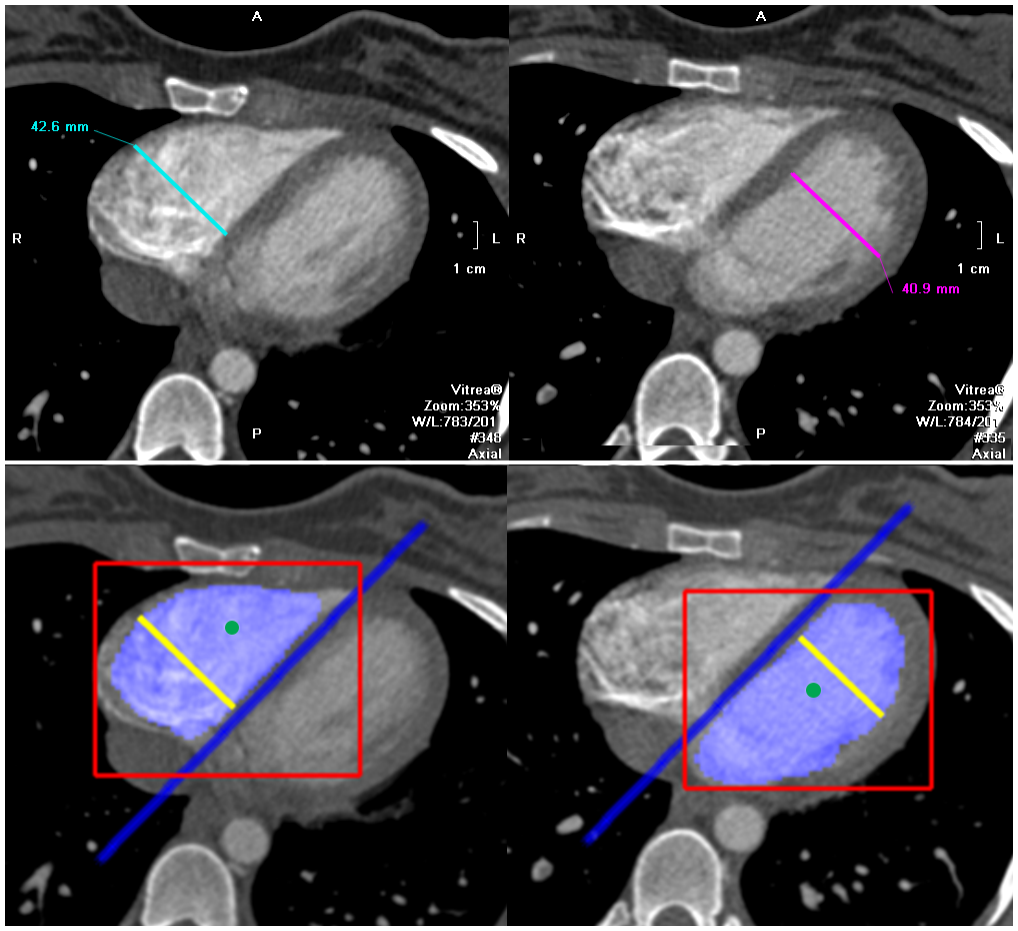
Axial images of a computed tomography pulmonary angiography of a 27 years old female with acute pulmonary embolism. Top row: manually estimated axial ventricular diameters. Bottom row: automatically estimated axial ventricular diameters and depiction of the different steps followed to compute them: ventricle detection (red boxes), seed positioning (green dots), interventricular septum estimation (blue line), ventricle segmentation (blue areas) and ventricular diameter estimation (yellow lines).

A completely automated algorithm (Fig 1) was designed to compute the RV/LV axial diameter ratio. An example of its output is shown in Fig 2. The CAD algorithm works in the following manner:

#### Ventricle Detection

A general machine learning based system for detecting objects in 2D images [18] is used to find the left and the right ventricles in each axial slice of the CTPA. The training set consists of 40 CTPAs obtained from the CAD-PE challenge (www.cad-pe.org) and is formed by axial images labeled with bounding boxes around left and right ventricles (positive training samples) and images were the heart is not visible (negatives training images). The output of the detection algorithm is a set of 2-dimensional bounding boxes per axial slice with an associated score representing its fitness to the model.

Only a subset of the axial bounding boxes truly represents the ventricles. To find such subset, we eliminate detections that are not coherent with the statistics on the location and size of the heart. Remaining detections across axial slices are linked with an unsupervised clustering algorithm based on the mean-shift technique [19]. Different clusters are then ranked according to a function based on the fitness scores of their detections and the number of detections in the cluster. Only the highest-ranking cluster is kept for each ventricle. Each axial bounding box in the surviving cluster is used to establish a seed point (green dots in Fig 2). A more detailed description of the ventricle detector can be found in [20].

#### Interventricular Septum Detection

The interventricular septum is modeled as a 3-dimensional plane and placed in the image by analyzing second-order image derivatives, combined to detect plane-like geometrical structures. We search for the maximum score of such detection algorithm in image locations between the seeds placed for the right and left ventricles. The plane-like detection algorithm also provides an orientation per voxel, which together with the location of the maximum score univocally defines the 3-dimensional plane, the estimated interventricular septum (blue line in Fig 2).

#### Ventricle Segmentation

A 3D level-set algorithm [21] is adapted to the automatic segmentation of the ventricles (blue areas in Fig 2). The level-set algorithm works by evolving a contour from the set of seed points, subject to a combination of image constraints, such as the local image curvature, the estimated interventricular septum, and edge priors. The parameters of the level-set method were fixed using the first 10 cases of the evaluation dataset. The same level-set parameters were used for all the CTPA images.

#### Caliper Positioning and Ratio Estimation

The ventricular diameters are estimated by measuring the length of the segmentation in each axial slice perpendicularly to the estimated interventricular septum. However, due to poor image contrast, ventricle segmentations can include parts of the atria. The shape of the segmentation is analyzed to find the atrioventricular valves by fitting a 10^th^ order polynomial to the contour of the segmentation and analyzing its roots and derivatives in each axial slice. Only calipers between the estimated atrioventricular valve and the apex are considered. The estimated ventricular diameter is the maximum of such calipers. Finally the RV/LV diameter ratio is calculated as the division of the estimated calipers. The automated RV and LV diameter calipers are visualized and reviewed by an expert a posteriori to determine whether they were correctly placed in the ventricles.

### Statistical Analysis

Statistical analyses were performed with JMP (JMP, Version 11. SAS Institute Inc., Cary, NC, USA). Summary statistics are computed for the diameters of the ventricles and for the RV/LV diameter ratio. Continuous variables are presented as mean and standard deviation. All 198 cases are used for the manually estimated measurements, while only calipers placed in the ventricles are considered for the automated method. When the analysis requires paired measurements, only manual measurements corresponding to correctly placed diameters by the automated method are taken into account.

Paired t-tests are used to analyze differences between manual and automated measurements. Bland-Altman plots and Pearson correlation coefficients are calculated to compare the automated and manual RV/LV diameter ratios and to assess inter-observer variability. The distributions of RV/LV diameter ratios for the different measurement methods categorized by the different clinical outcomes are summarized by their mean and standard deviation.

The predictive value of the manually and the automatically computed RV/LV diameter ratio is established using logistic regression models adjusted for age and sex. Likelihood ratio tests are performed to establish the relevance of each variable in the prediction of clinical outcome.

Receiver-operating characteristic (ROC) analysis compare the performance of such models for predicting (a) PE-related death in 30 days and (b) a composite outcome of PE-related 30-day mortality or the need for one or more intensive therapies. Areas under the curves (AUC) are used for model comparison. Statistical significance between models is established using Chi-Square goodness of fit tests.

### Software Implementation

**Fig 3:**
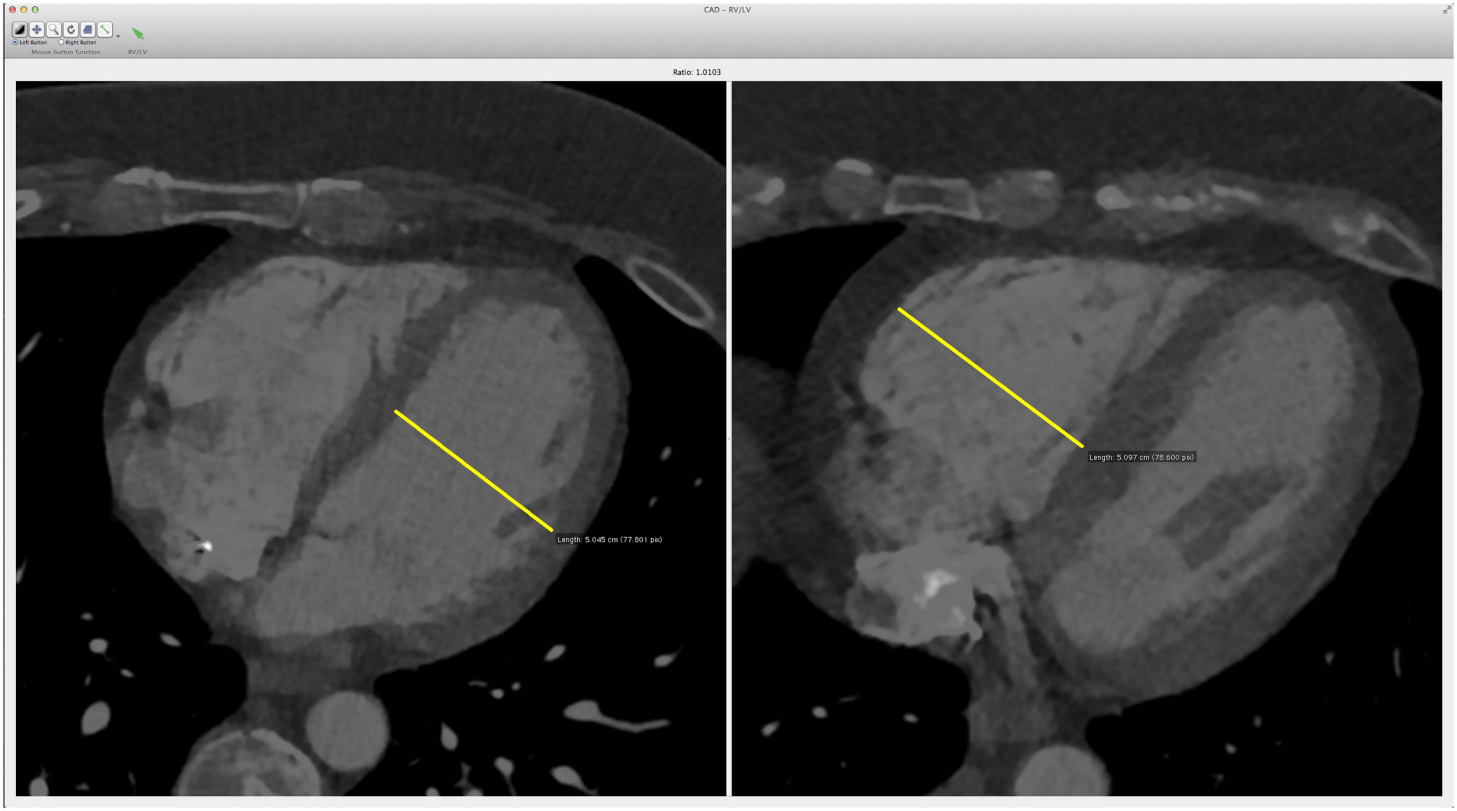
Interface designed to visualize the automatically computed axial ventricular diameters. The system automatically locates the diameters and adjusts the viewing window to maximize contrast. The RV/LV diameter ratio is shown between the two images.

The algorithm is implemented in Matlab (The Mathworks Inc. Natick, Massachusetts, U.S.A.) and integrated with the Osirix (Pixmeo, Geneva, Switzerland) radiology workstation. By selecting the case name, the radiologist is forwarded to an interface (Fig 3) that shows the computed axial right ventricular and left ventricular diameters.

## Results

The CAD system computed automatically the axial RV/LV diameter ratio for all the CTPA cases forwarded to the modified Osirix radiology workstation. The mean time needed to compute the RV/LV diameter was 4 minutes per case; this process is run in the background, prior to the analysis of the case, and does not require input from the radiologist. The output is reviewed with the interface (Fig 3) to decide if the calipers are properly placed in the ventricles. It takes roughly two seconds to make this decision.

The proposed CAD system correctly detected both ventricles in 96% (190/198) of the CTPA studies. Both RV and LV diameter calipers were placed in the corresponding ventricles in 92.4% (183/198) studies. Errors on ventricle detection or caliper positioning were due to poor contrast on the right ventricle (mean attenuation < 150 HU, n = 6); patient positioning (n = 3); exceptionally high contrast levels in the right ventricle (n = 2); abnormal ventricle shape (n = 1); and unknown reasons (n = 3).

### Comparison against manually computed RV/LV ratio

**Fig 4:**
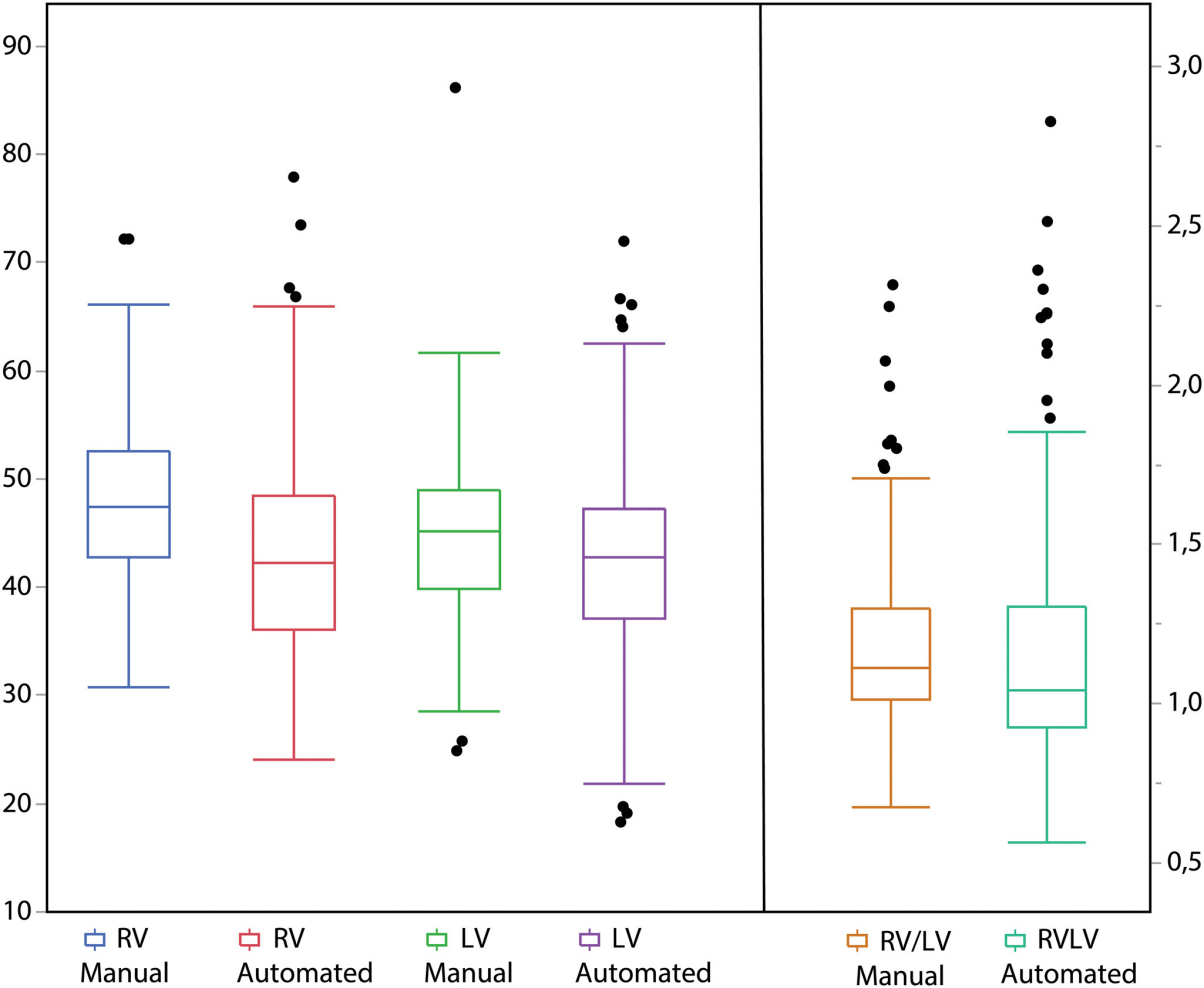
Tukey outlier box plot of manually and automated extracted ventricular diameters and diameter ratios. The box is placed at the median of the distribution. Top and bottom limits of the boxes represent 1^st^ and 3^rd^ quartiles of the data respectively. Whiskers length is 1.5 inter-quartile distances. Individual dots are considered outliers. While both RV and LV are underestimated by the automated method, the manually and automated estimated ratios are not significantly different, as shown in Table 1.

Table 1 compares summary statistics of the automated and manually estimated diameters and ratios. Tukey plots of the different measurements are shown in Fig 4. The CAD system underestimates the RV diameter by a mean value of 4.8±6.4 mm and the LV diameter by a mean of 2.2±8.1 mm, both with p < 0.001. The comparison of the manually estimated ratio and the automatically estimated one is small and not statistically significant (difference 0.03±0.22, p = 0.08). A qualitative example is shown in Fig 3.

**Table 1:** Mean and standard deviation values for the automated and manual axial ventricular diameter estimations and RV/LV diameter ratios.

	Manual	CAD System	Difference	p-value
RV (mm)	47.9 ±8.1	43.2 ±9.3	4.8 ±6.4	< 0.001
LV (mm)	44.6 ±7.4	42.3 ±9.5	2.2 ±8.1	< 0.001
RV/LV	1.10 ±0.27	1.08 ±0.37	0.03±0.22	0.08

P-values are obtained using paired t-tests.

**Fig 5:**
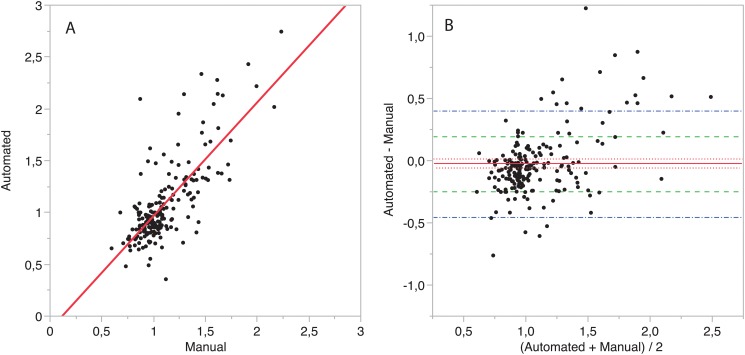
Comparison of automated and manually computed RV/LV diameter ratios. A) Correlation plot. A linear model is fitted to the data. The intercept’s value is -0.16, the slope is 1.11, Person’s correlation coefficient is R = 0.81, 95% CI [0.76–0.86]. B) Bland-Altman comparison. The mean difference is -0.023 (95% CI [-0.061 0.003], p = 0.08), is depicted with a red line and its 95% CI as dotted red lines. Dot-dashed blue line: 95% limits of agreement of manual vs. automated measurement. Dashed green line: 95% CI limits of agreement between two expert readers. 72.1% (132/183) cases are within the limits of inter-reader variability.

A linear correlation plot of the automated versus the manual diameter ratio is shown in Fig 5A. The linear fit has a small intercept (-0.16) and a slope of 1.11 (p < 0.001). Pearson’s correlation coefficient between manual and automated diameter ratios is 0.81, 95% CI [0.79 0.86].

Bland-Altman analysis of manual and automated methods is shown in Fig 5B. The mean difference is very small: -0.023 (95% CI [-0.059 0.013]). The 95% limits of agreement of the difference of the automatically computed RV/LV diameter ratio and the manual one were -0.47 and 0.46. 72.1% (132/183) cases are within the 95% limits of agreement between two independent readers.

Inter-observer variability, assessed in the 30 cases with dual readings, had a Pearson correlation coefficient of 0.73, 95% CI [0.50–0.86]. The second reviewer estimates larger diameters than the first reviewer, by a mean value of 4.13±4.5 mm for the RV and 6.08±4.9 mm for the LV, both with p < 0.001. RV/LV diameter ratios obtained by the two reviewers are not statistically significantly different (difference 0.03±0.11, p = 0.10). For the nine patients were the axial RV/LV diameter ratio measurement time was documented, the mean time was 106±26 seconds.

### Prognostic value of the automated RV/LV axial diameter ratio

The automated RV/LV diameter ratio has similar predictive value to manually estimated diameter ratios for both clinical outcomes, as shown by the following aggregated statistics and ROC analysis.

Table 2 summarizes the mean values of the automated and manual diameter ratios. Patients that die from PE have higher mean RV/LV diameter ratio, both using manual (1.31 vs. 1.08, p = 0.0001) and automatic ratio estimation (1.32 vs. 1.05, p = 0.0018). Patients who die from PE or those who require intensive therapies also have higher average RV/LV diameter ratio for both manual (1.32 vs. 1.05, p < 0.0001) and automated (1.36 vs. 1.02, p < 0.0001) measurements.

**Table 2:** Mean and standard deviation values of the RV/LV diameter ratios computed manually and automatically for two clinical outcomes.

	30 days PE-Related Death			30 days PE-Related Death or Intensive Therapies		
	Positive	Negative	p-value	Positive	Negative	p-value
Manual	1.31± 0.35	1.08± 0.25	<0.0001	1.32± 0.32	1.05± 0.23	< 0.0001
CAD System	1.32± 0.52	1.05± 0.34	0.0018	1.36± 0.49	1.02± 0.32	< 0.0001

P-values are obtained using paired t-tests.

**Fig 6:**
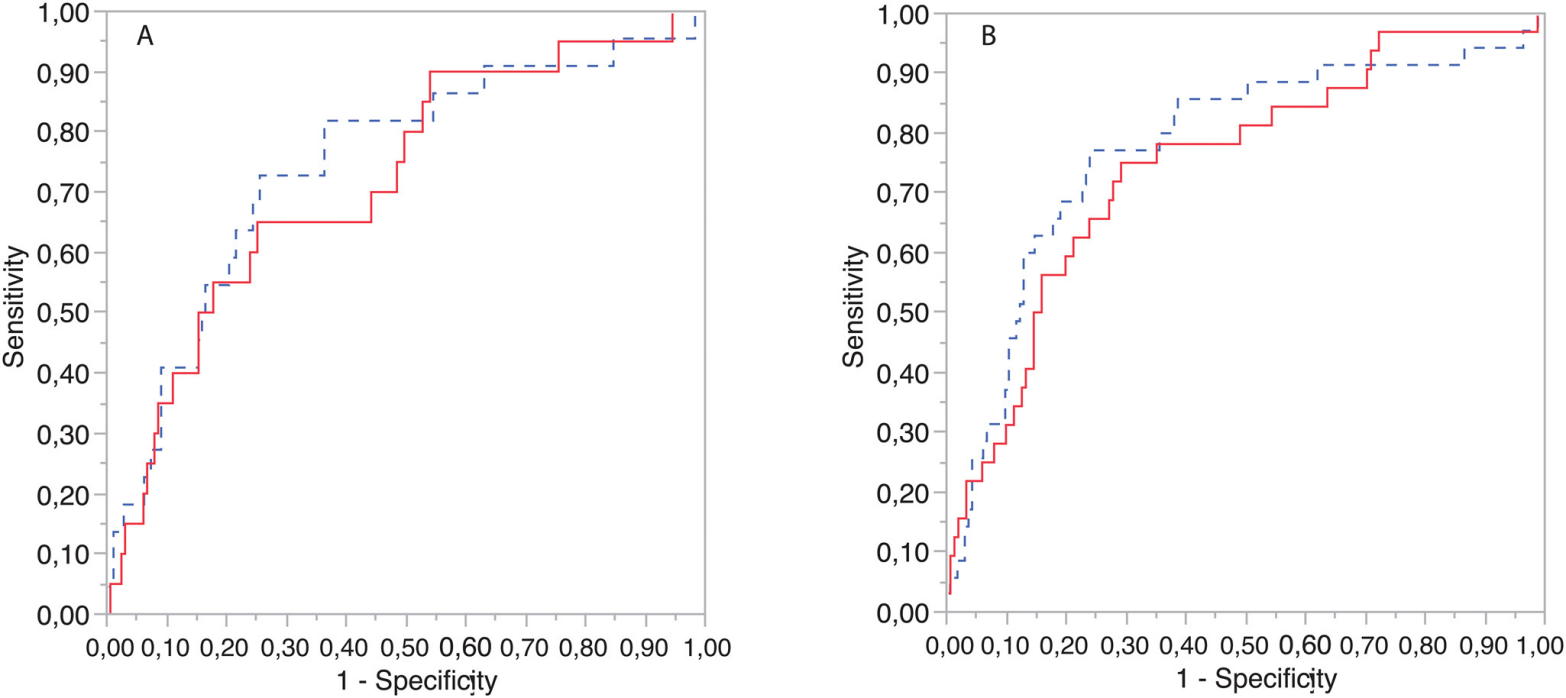
Prediction of clinical outcome. ROCs curve for the manual (dashed blue line) and automated (red line) logistic regression models when used to predict: A) 30 days PE-related mortality and B) 30 days PE-related mortality or the need for intensive therapies. Areas under the curve are 0.75 for the manual method and 0.72 for the automated method for Fig 6A and 0.78 for the manual method and 0.75 for the automated method in Fig 6B.

In the predictive model for 30-days PE-related mortality, RV/LV diameter ratios are significant predictors after adjusting for age and gender, with p-values of 0.0005 for the manual method and 0.008 for the CAD system. Fig 6A shows ROC curves of the manual and automated logistic regression models for 30-days PE-related mortality as clinical outcome. AUC analysis shows that manual and automated RV/LV diameter ratios have similar predictive values of 0.75 (95% CI [0.61 0.85]) and 0.72 (95% CI [0.58 0.82]) respectively. The Chi-Square test does not establish that the predictive values of both models are different (p = 0.408).

Similarly, RV/LV diameter ratios are significant predictors of 30-days PE-related death or the need of intensive therapies after adjusting for age and gender, with p < 0.0001 for both manual and automatically estimated ratios. Fig 6B shows ROC curves of the manual and automated logistic regression models for this clinical outcome. AUC analysis shows that manual and automated RV/LV diameter ratios have similar predictive values of 0.78 (95% CI [0.67 0.86]) and 0.75 (95% CI [0.64 0.83]) respectively. The Chi-Square test cannot establish that the two predictive models are different (p = 0.28). Areas under the curves and p-values are summarized in Table 3.

**Table 3:** Area under the curve (AUC) comparison for the different methods and clinical outcomes. P-values are obtained with Chi-Square Goodness of Fit Tests.

	30 days PE-related death (95% CI)	30 days PE-Related Death or Intensive Therapies (95% CI)
Manual	0.75 [0.61 0.85]	0.78 [0.67 0.86]
CAD System	0.72 [0.58 0.82]	0.75 [0.64 0.83]
Difference in AUC	0.027 [-0.09 0.04]	0.031 [-0.09 0.03]
P > ChiSq	0.408	0.280

## Discussion

Prognosis data from CTPA is a valuable component in the decision of whether or not to manage an acute PE patient with intensive therapies [22]. Although the RV/LV ratio has been shown to be a good prognostic biomarker for patients suffering from acute PE, it is likely that this metric is not universally reported, probably because of the significant inter-reader variability and the moderate effort needed to perform and report the measurements. While recent research has focused on subjective evaluation of right ventricular enlargement [16], the performance will be related to the experience of the radiologist.

The proposed CAD system automatically provides an objective metric of axial RV/LV diameter ratio that has high accuracy when compared to manual measurements. There is no statistically significant difference between the reference standard measurements made manually and those that the proposed software outputs automatically. The correlation coefficient of the automated method with respect to the manual one is comparable to the correlation coefficient obtained for inter-reader variability and inter-reader variability reported in other studies [15]. The difference in prognostic value of the automatically and manually computed RV/LV diameter ratios is not statically significant for the two clinical outcomes studied: 30-day PE-related mortality and 30-day PE-related mortality or the use of intensive therapies.

It is relevant to note that the proposed methodology deals with important challenges related to the nature of the CTPA datasets. The heart in CTPA studies can present severe motion artifacts [23]. Variability on the timing of the contrast bolus can heavily change the contrast enhancement within the right ventricle. Trabeculae and other endocardial structures show low contrast with respect to the myocardium. The presented algorithm has proven robust to such artifacts in most of the cases analyzed (92.4%), failing only on the most extreme cases. In practice, these errors can be readily identified by the radiologist before reporting.

The goal of this study is to present software to report the RV/LV diameter ratio automatically using the standard clinical data and to retrospectively evaluate its accuracy using a cohort of patients for whom there is a comprehensive, published database of clinical outcomes. The presented CAD system reports measurements on axial images, and thus the study does not include volumetric measurements or 4-chamber reformatted images. This has been a design choice that prioritizes easy supervision, as axial RV/LV diameter can be reviewed without image reformatting and, moreover, the prognostic value of measurements derived from the axial images alone has not been shown to be inferior to such of four-chamber reformatted computed diameter ratios [12].

We acknowledge several limitations. First, the study design did not include transthoracic echocardiography data. While such data were not available, it has been shown that echocardiography RV diameter estimations and CT RV/LV diameter ratios have a moderate Spearman correlation coefficient [24] and similar 30-days PE-related mortality prognostic significance [25]. The manual CTPA measurements were considered an ideal reference standard since, at the time of CTPA interpretation, it is unlikely that the radiologist will have echocardiography data with a comparable RV/LV diameter ratio. Second, this study focused in the evaluation of the automatically computed diameter ratios and does not explore the possibility of correcting automatically placed calipers. The development and analysis of such functionality is currently under investigation and is beyond the scope of the current project. Third, ensembles of biomarkers have been proven better risk stratification predictors than ensembles of biomarkers and manually computed axial RV/LV diameter ratio [26]; it would be interesting to replicate such study with the automatically computed ratios. Fourth, we did not exclude other causes of pulmonary hypertension, which may have lowered the prediction accuracy of PE-related death by RV/LV diameter ratio. However, these data would not alter the study conclusion.

This work exemplifies a revisited paradigm related to the fully automatic computation of image-based biomarkers from standard clinical imaging datasets. In the growing scenario of digital data availability, increasing radiology department burden, and initiatives to transform big data to clinical knowledge [27], the automatic computation of image based biomarkers is gaining substantial traction. In these setups, robustness and reproducibility must be guaranteed and the equivalence of the derived clinical outcomes should be confirmed. This project follows this approach, devoting the efforts to enable automatic robust computations as well as dealing with a challenging and time consuming task in the evaluation of prognosis of PE patients. The extension of these approach to other fields of pulmonary and or vascular imaging constitutes a desirable trend as it has been recently and successfully approached by other investigators [28,29].

## Conclusions

We present a new automated CAD system that inputs DICOM CTPA images and reports a measurement of the right ventricular to left ventricular diameter ratios comparable to manual assessment by a radiologist. The CAD system has high accuracy and high prognostic value with respect to two clinical outcomes: 30-days PE related death or 30-days PE related death or the need of intensive therapies. Future studies will test the integration of this software into clinical workflow.
